# Supplementary material for: Atomic order of rare earth ions in a complex oxide: a path to magnetotaxial anisotropy
Source: Nat Commun. 2024 Jun 14;15:5083. doi: 10.1038/s41467-024-49398-4 (PMC11178793; doi:10.1038/s41467-024-49398-4)
Supplement: Supplementary file 1 — Supplementary Information [file 41467_2024_49398_MOESM1_ESM.pdf]

Atomic order of rare earth ions in a complex oxide: a path to magnetotaxial anisotropy

Allison C. Kaczmarek[1], Ethan R. Rosenberg[1,2*], Yixuan Song[1], Kevin Ye[1], Gavin A. Winter[1], Aubrey N. Penn[3], Rafael Gomez-Bombarelli[1], Geoffrey S.D. Beach[1], and Caroline A. Ross[1]*

[1] *Department of Materials Science and Engineering, Massachusetts Institute of Technology, Cambridge, Massachusetts, 02139, USA*

[2] *Lawrence Livermore National Laboratory, Livermore, California, 94550, USA*

[3] *MIT.nano, Massachusetts Institute of Technology, Cambridge, Massachusetts, 02139, USA*

**These authors contributed equally to the work.*

Corresponding Author: Allison C. Kaczmarek; kacz@mit.edu

Supplementary Information

Contents **Page**

Note 1: *Callen's theory of growth-induced anisotropy (GIA)* 2

Note 2: *Strain calculation and additional high-resolution x-ray diffractograms* 8

Note 3: *Vibrating sample magnetometry background subtraction and error propagation* 11

Note 4: *Spin Hall magnetoresistance and anisotropy calculations* 13

Note 5: *Derivation of uniaxial anisotropy from three tilted anisotropies* 15

Note 6: *Scanning transmission electron microscopy (STEM)* 18

Note 7: *Density Functional Theory (DFT) Calculations* 25

Note 8. *Simulations of X-ray Diffraction (XRD) from ordered garnets* 29

Note 9. *Relationship between XRD and STEM data for verification of cation order* 32

References 35

Supplementary Note 1: *Callen's theory of growth-induced anisotropy (GIA)*

This description of the site preference model has been summarized from the work of Herbert Callen and Andrew Eschenfelder.[1–4]

(1) GIA occurs in complex oxides in which spin-orbit coupling (SOC) makes the magnetic properties sensitive to the type and arrangement of electrons in neighboring ions. Strong SOC also explains the existence of magnetocrystalline anisotropy.[5]

(2) GIA originates from the rare-earth ion sites (*c* sites) due to the different orientations of the coordination dodecahedra within the unit cell with respect to neighboring magnetic cations (Fe^{3+} and RE^{3+}). At the growth surface, differently oriented *c* sites present different coordination constellations to incoming cations. Each unit cell has 24 *c* sites comprising 12 different orientations. The 12 types of sites can be obtained by geometrical operations on one site, which will be referred to as X1.

   - X2 are obtained by inversion, X3 obtained by reflection in the x-y or x-z plane, and X4 by a combination of the two reflections.
   - Y1, Y2, Y3, Y4, Z1, Z2, Z3, Z4 are obtained by cyclic permutations of the axes on the operations to generate the X family (e.g. switch x for z axis and repeat the above procedure).

Table S1 lists the relative coordinates of each of the dodecahedral sites in the cubic garnet unit cell. A .cif file of the unit cell with labeled sites is also available upon request.

Table S1. *Fractional coordinates of labelled dodecahedral sites in the garnet unit cell*

	x/a	y/b	z/c
X1	0.25	0.375	0.5
X1	0.75	0.875	0
X2	0.25	0.125	0
X2	0.75	0.625	0.5
X3	0.25	0.875	0.5
X3	0.75	0.375	0
X4	0.25	0.625	0
X4	0.75	0.125	0.5
Y1	0.375	0.5	0.25
Y1	0.875	0	0.75
Y2	0.125	0	0.25
Y2	0.625	0.5	0.75
Y3	0.375	0	0.75
Y3	0.875	0.5	0.25
Y4	0.125	0.5	0.75
Y4	0.625	0	0.25
Z1	0	0.25	0.625
Z1	0.5	0.75	0.125
Z2	0	0.75	0.375
Z2	0.5	0.25	0.875
Z3	0	0.256	0.125
Z3	0.5	0.75	0.625
Z4	0	0.75	0.875
Z4	0.5	0.25	0.375

(3) Contributions to magnetic anisotropy from each site is found by summing $\cos^2(\vec{a}\vec{r}_{ij})$ for each of its nearest magnetic neighbors, where $\vec{r}_{ij}$ is the unit vector between the two ions and $\vec{a}$ is the magnetization of the neighboring ion. Summing for all RE sites we get the following equation for magnetic energy of a garnet with mixed A and B RE ions, relative to the unmixed A garnet:

$$\epsilon = C_0[N_x\alpha_1^2 + N_y\alpha_2^2 + N_z\alpha_3^2] - 16C_3[N_{\bar{x}}\alpha_2\alpha_3 + N_{\bar{y}}\alpha_3\alpha_1 + N_{\bar{z}}\alpha_1\alpha_2] \quad (1)$$

where C_0 and C_3 are constants and α are direction cosines. N_i are the number of B ions in the X, Y, and Z sites, and:

$$N_x \equiv N_{x1} + N_{x2} + N_{x3} + N_{x4} \tag{2}$$

$$N_{\bar{x}} \equiv N_{x1} + N_{x2} - N_{x3} - N_{x4} \tag{3}$$

$$N_x + N_y + N_z = 3N_0x \tag{4}$$

where N_0 is the number of formula units of $(A_{1-x}B_x)_3Fe_5O_{12}$ in the crystal (x is the fraction of B ions in the crystal).

(4) For different growth faces, *c* sites fall into symmetrically inequivalent categories according to how the site symmetries are reduced at the surface. The anisotropy energy can be simplified for each case in which a B ion is substituted in an A site. The *c* sites are reduced into groups α, β, γ, δ of degenerate sites according to the symmetry of the neighbors around each site at the growth surface. The sets of equivalent sites and the simplified expressions for energy for several growth surfaces are:

- (110) Sites fall into three groups, with 2/3 of them in the α group and 1/6 in each of the β and γ groups.

$$\alpha: N_{X1} = N_{X3} = N_{Y2} = N_{Y4}, N_{X2} = N_{X4} = N_{Y1} = N_{Y3} \tag{5}$$

$$\beta: N_{Z1} = N_{Z2} \tag{6}$$

$$\gamma: N_{Z3} = N_{Z4} \tag{7}$$

$$\epsilon = \frac{3}{2}C_0\left(N_z - \frac{1}{3}N\right)\alpha_3^2 - 16C_3N_{\bar{z}}\alpha_1\alpha_2 \tag{8}$$

- (112) Sites fall into four groups, with 1/4 of them in each group.

$$\alpha: N_{X1} = N_{Y2} \tag{9}$$

$$\beta: N_{X2} = N_{Y2} \tag{10}$$

$$\gamma: N_{X3} = N_{Y4} \tag{11}$$

$$\delta: N_{X4} = N_{Y3} \tag{12}$$

$$\epsilon = \frac{1}{2}C_0(3N_z - N)\alpha_3^2 - 16C_3[N_{\bar{x}}\alpha_3(\alpha_1 + \alpha_2) + N_{\bar{z}}\alpha_1\alpha_2] \tag{13}$$

- (111) Sites fall into two groups, with 1/2 of them in each group.

$$\alpha: N_{X1} = N_{X2} = N_{Y1} = N_{Y2} = N_{Z1} = N_{Z2} \tag{14}$$

β: $N_{X3} = N_{X4} = N_{Y3} = N_{Y4} = N_{Z3} = N_{Z4}$ (15)

$$\epsilon = -8C_3N_{\bar{x}}(\alpha_1 + \alpha_2 + \alpha_3)^2 = -24C_3N_{\bar{x}}\alpha_{111}^2 \quad (16)$$

- (001) Sites fall into two groups, with 2/3 of them in the α group and 1/3 in the β group.

α: $N_{X1} = N_{X2} = N_{X3} = N_{X4} = N_{Y1} = N_{Y2} = N_{Y3} = N_{Y4}$ (17)

β: $N_{Z1} = N_{Z2} = N_{Z3} = N_{Z4}$ (18)

$$\epsilon = \frac{3}{2}C_0\left(N_z \quad -\frac{1}{3}N\right)\alpha_3^2 \quad (19)$$

It is interesting to note that for the (111) and (001) cases, the magnetic anisotropy is uniaxial with the unique axis perpendicular to the growth face.

(5) Concentration (x) dependence – In the (110) ordering scheme (as an example), A and B ions would have different "sticking coefficients" in each of the α, β, and γ sites.
Then, each site would contain some fraction of B ions: for the α sites

$$p_\alpha^x \equiv \frac{xp_\alpha}{(1-x)(1-p_\alpha)+xp_\alpha} \quad (20)$$

and analogous expressions for the other sites.
At low concentrations, the energy equation can be simplified and approximated for weak site preference to depend on concentration quadratically, scaling as $x(1\text{-}x)$.

The symmetry reduction of the unit cell for ordering on different growth surfaces is visualized in Fig. S1. As the symmetry of the growth surface is reduced from (111) → (110) → (112), the dodecahedral sites are split into 2, 3, and 4 degenerate site groups, as described above. The symmetry of the unit cells of resulting crystalline materials grown on these surfaces have lower symmetry than the unordered cubic cell (space group: $Ia\bar{3}d$). Considering a garnet film which includes all the symmetry variants of the atomic ordering, the space groups of these ordered cells are summarized in Table S2.

These site-ordered, symmetry-reduced structures are not conventional superlattices but they represent a 3D ordering based on the geometrical orientation of the 24 dodecahedral sites in the unit cell. The ordered structures are formed during the growth of these thin films by the preference of arriving RE ions to order into inequivalent dodecahedral sites due to steric factors.

Once formed, the site-ordering is highly stable at ambient conditions, and prior work has shown that the growth-induced anisotropy is robust up to high temperatures[4], which indicates that the growth-induced order is retained to high temperatures. The order and the resulting anisotropy can be lost by a sufficiently high temperature anneal (e.g. >600˚C) to allow for RE diffusion within the structure.

Table S2. *Crystallographic space groups of cation-ordered garnets.*

**Order Scheme**	**Crystallographic Space group**	**Crystal system**
No order	$Ia\bar{3}d$ (230)	Cubic
(111) growth surface	R3c (167)	Trigonal
(110) growth surface	Fddd (70)	Orthorhombic
(112) growth surface	C2/c (15)	Monoclinic

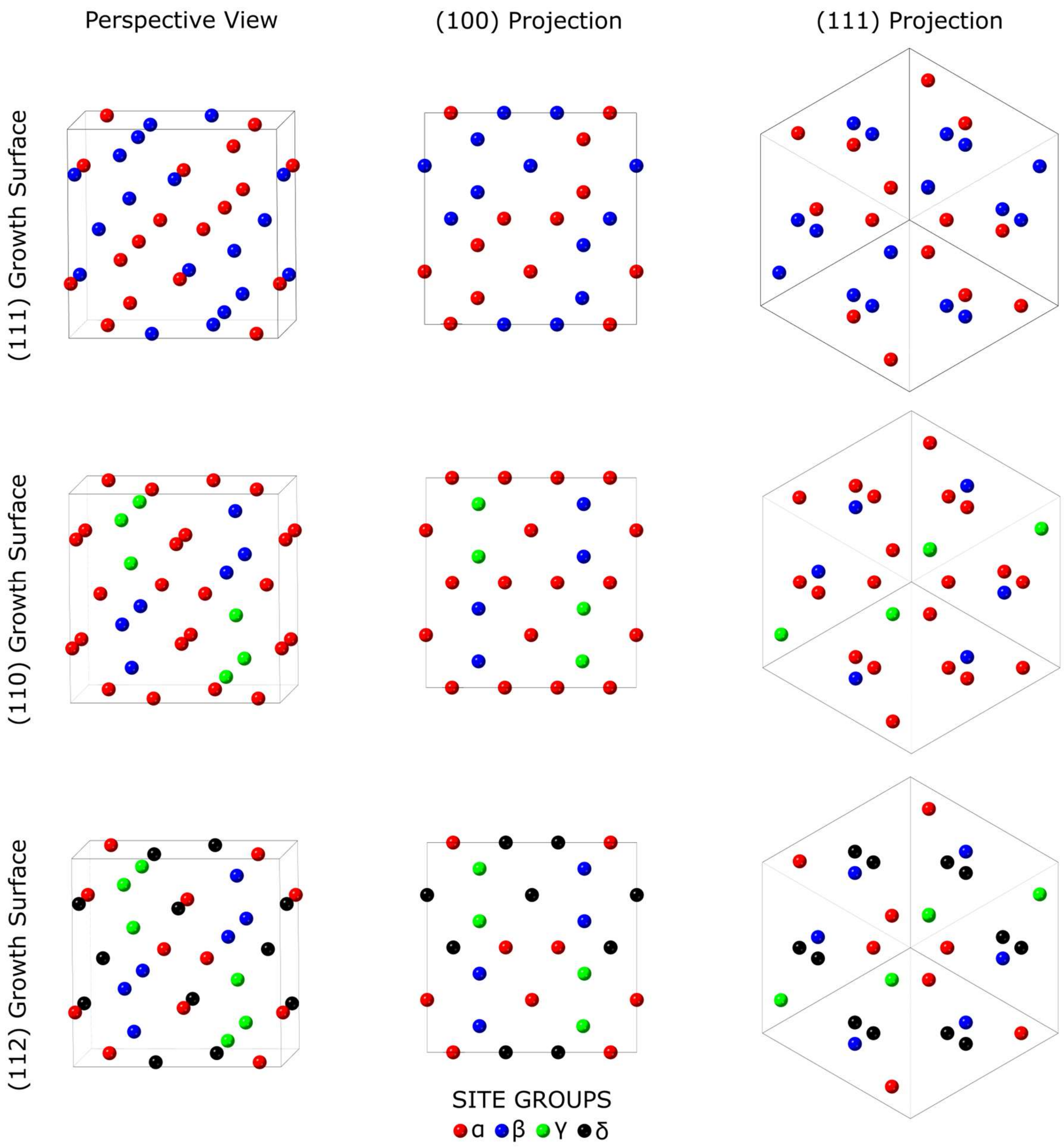

Fig. S1. Order schemes and inequivalent dodecahedral site groups for the [111], [110], and [112] growth directions.

Supplementary Note 2*: Strain calculation and additional high-resolution x-ray diffractograms*

Reciprocal space mapping of the (642)+ reflection shows that the thickest film with the largest lattice mismatch (EuIG, 42 nm) is fully strained within the plane of the film, since the film and substrate peaks have the same qx value, as shown in Fig. S2(a). Therefore, it is assumed in all calculations that thinner films or those with less lattice mismatch will also be fully strained. The out-of-film plane spacing, related to 2θ of the (444) reflection (Fig. S2(b)), can be used to calculate the lattice parameter and strain of the film using expressions for a rhombohedral distortion.

The presence of Laue oscillations on the film peak as well as low spread in the rocking curve, as shown in Fig. S2(c), indicate high crystalline quality, low degree of mosaic spread, and planar top and bottom interfaces. Mixed garnet films of all compositions show similar rocking curve widths, indicating similar quality (Table S3). Fringes also allow us to fit thickness of the film, analogously to Kiessig fringes in thin film reflectivity. Fig. S2(d) shows that only garnet peaks corresponding to the out of plane lattice direction are present over a wide range of angles, confirming the phase purity of the films.

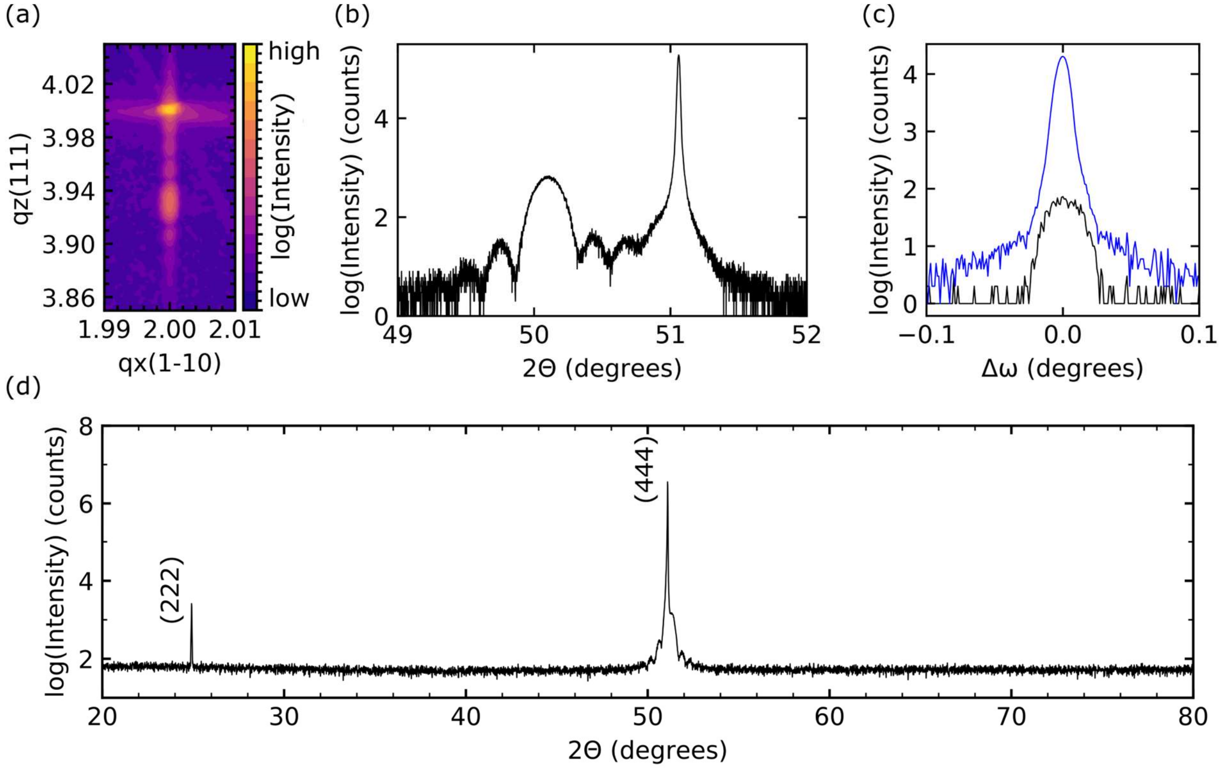

Fig. S2. Thin film XRD characterization. (a) (642)+ reciprocal space map of 42 nm EuIG on GGG. (b) (444) coupled scan of (111) EuIG on GGG. (c) Rocking curves for the film (EuIG) and substrate (GGG), in black and blue, respectively. (d) Coupled symmetric scans over a 2θ range of 20 – 80° showing only garnet peaks for the mixed Eu:Tm garnet film with x = 19.6% on (111) GGG.

With the Globalfit software, the film peak is fitted assuming the film is fully relaxed, as shown in Fig. S3(a), although reciprocal space mapping shows that films are fully strained. This fit gives us the relaxed lattice parameter, and hence d_f^{444} , the lattice spacing in the out of plane direction of the film (Fig. S3(b)):

$$d_{444}^{f} = \sqrt{\left(a_{100}^{f}\right)^2 + \left(\sqrt{2}a_{100}^{f}\right)^2} \tag{21}$$

Using similar triangles, we obtain the in-plane substrate spacing (Fig. S3(c)):

$$d = \sqrt{\frac{2}{3}}a_s \tag{22}$$

and finally by the rhombohedral relation (Fig. S3(d)):

$$h = d_{444}^{f}/3 \tag{23}$$

we can determine shear lattice strain, corner angle, and lattice constant of the fully strained film (Fig. S3(d)):

$$\beta = 2\sin^{-1}\left(\frac{\sqrt{2}}{2}\frac{a_s}{a_0^f}\right) \tag{24}$$

$$a_0^f = \sqrt{d^2 + h^2} \tag{25}$$

The GGG lattice parameter used in fitting is a_s = 1.2376 nm. We calculate film lattice parameter and strain geometrically (without literature values for stiffness) since we can assume that the films are fully strained to match the substrate in-plane lattice parameter.

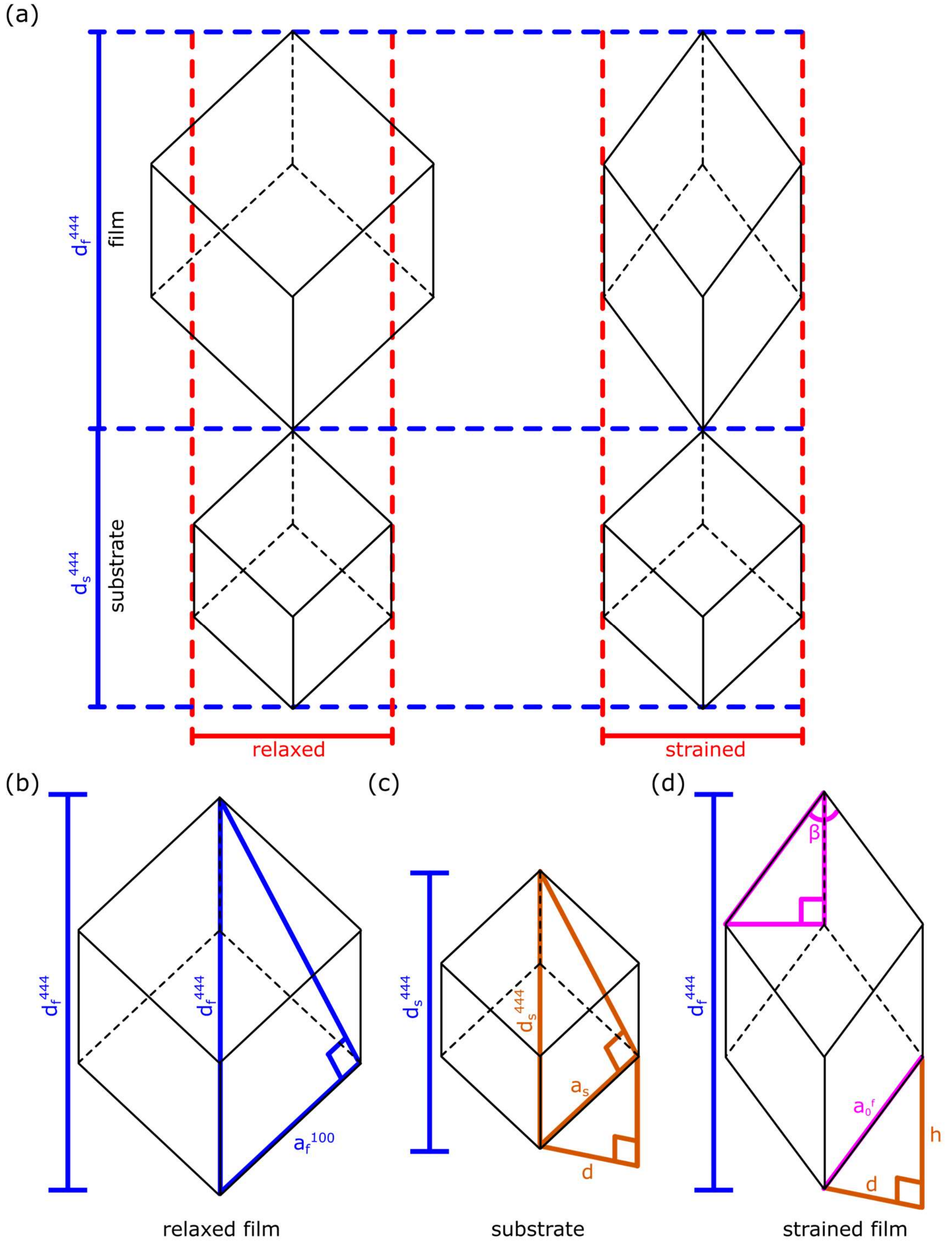

Fig. S3. Geometric representation of a strained (111) unit cell on a substrate. (a) Schematic of fully relaxed and fully strained films with the same out of plane lattice spacing. (b) Geometric relation between out of plane spacing and relaxed lattice parameter. (c) Geometric relation for in plane spacing, d. (d) Rhombohedral relation for strained film.

The lattice parameter can be used to determine the composition by the lever rule based on the strained lattice parameters of the end members, EuIG and TmIG. From the fitted thickness, the growth rates of the EuIG and TmIG are found to be 239 shots per nm and 614 shots per nm, respectively. Table S3 outlines the shot ratios, compositions, thicknesses, and strains of the REIG films, and the rocking curve widths.

Table S3. *Summary of structural properties for REIG films*

Shot ratio (Eu:Tm) per cycle	Fraction of EuIG, (x)	Thickness, t, (nm)	Film lattice parameter, a_f, (nm)	Corner angle, β, (°)	Full width-half maximum values of the rocking curve peak (°)
0:35	0	16.3	1.2337	90.36	0.024
5:30	0.196	25.3	1.2360	90.15	0.024
8:27	0.503	26.6	1.2394	89.83	0.021
12:23	0.613	28.1	1.2407	89.72	0.025
27:8	0.905	40.7	1.2440	89.41	0.021
35:0	1	41.8	1.2450	89.32	0.027

Supplementary Note 3: *Vibrating sample magnetometry background subtraction and error propagation*

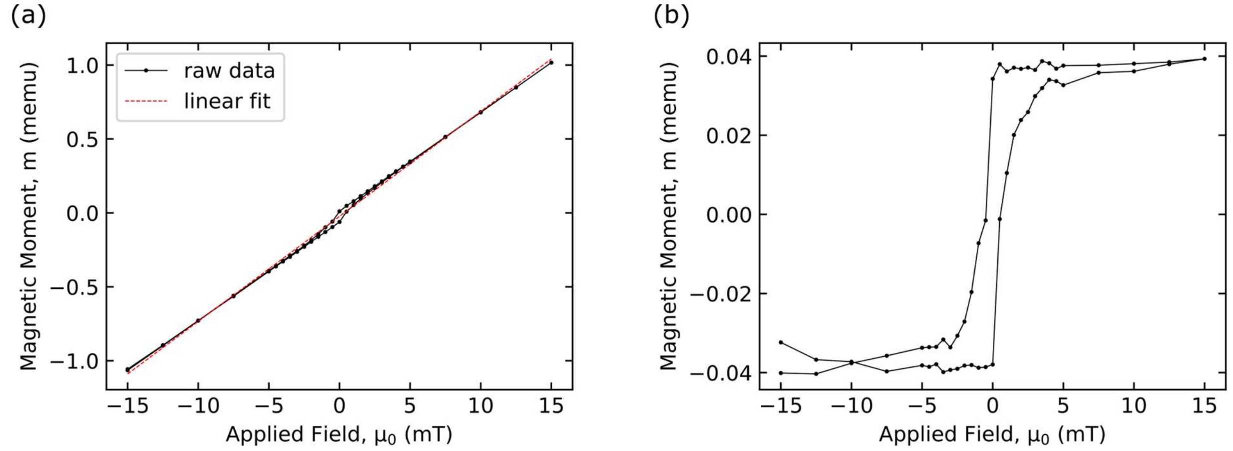

Fig. S4. VSM analysis. (a) Raw VSM signal with linear fit. (b) Background-corrected VSM signal.

For the determination of M_s for films grown on GGG by magnetometry, it is well known that the paramagnetic signal from GGG must be subtracted, or the substrate must be thinned down to reduce this signal.[6] Fig. S4 shows the VSM hysteresis loop for TmIG before and after linear background subtraction of each of the four tails (for a symmetric full loop).

The error associated with measuring the M_s of a given film can be estimated by the following contributions. Sample mounting error ($\chi \pm \delta\chi$, units of emu/V) is estimated from the sample standard deviation of measured VSM calibration factors. Fitting error ($V \pm \delta V$, units of V) is the standard error for the linear fit of the saturated branches of the hysteresis loops. The total error, ($\mu \pm \delta\mu$, units of emu), is thus the combination of these two errors:

$$(\chi \pm \delta\chi)(V \pm \delta V) = \chi V\left(1 \pm \sqrt{\left(\frac{\delta\chi}{\chi}\right)^2 + \left(\frac{\delta V}{V}\right)^2}\right) \tag{26}$$

This analysis is only applicable for easy axis loops (in the field range of 0.02 T). Beyond ~ 0.2 T, GGG has a non-linear background, so it is difficult to determine the hard axis saturation, which according to SMR measurements occurs for these garnets at most around 0.34 T. This is why only easy axis loops are reported. Table S4 reports the coercivities of the unpatterned films from the easy axis loops.

Table S4*: Coercivity of the unpatterned films.*

Shot ratio (Eu:Tm) per cycle	Hc (mT)
0:35	11.00
5:30	3.53
8:27	7.70
12:23	38.44
27:8	3.36
35:0	10.52

***Supplementary Note 4**: Spin Hall magnetoresistance and anisotropy calculations*

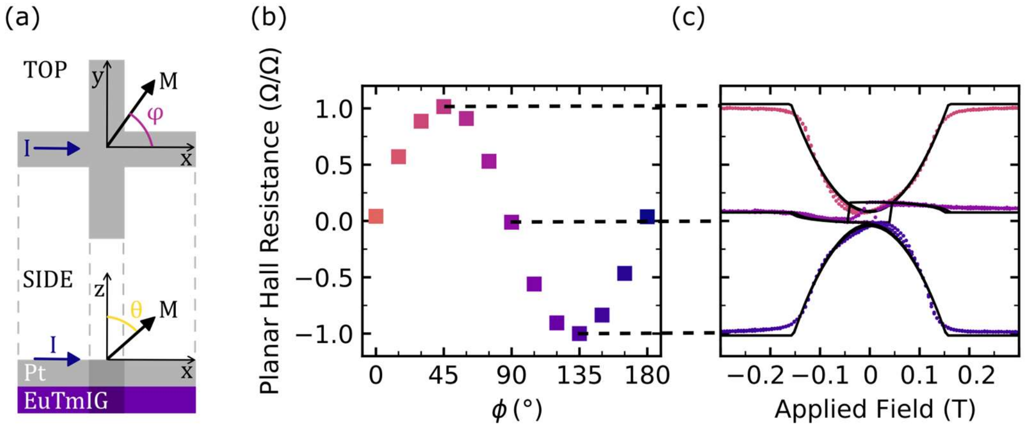

Fig. S5. Spin Hall Magnetoresistance Measurements. (a) SMR device and measurement geometry. (b) Planar transverse hall resistance as a function of in plane field angle. (c) Representative SMR measurements showing the in-plane saturation field, which is related to magnetic anisotropy.

To determine the magnetic anisotropy energy (MAE) of each PMA $(Eu_xTm_{1-x})_3IG$ sample, we take advantage of spin Hall magnetoresistance measurements[7]. A thin (4 nm) layer of Pt was deposited on the garnet films which is used as a spin current detector, giving a signal that scales with the magnetization of the film when a current is applied to the Pt layer. The Hall cross and measurement geometries are described in Fig. S5(a). For each PMA sample, an in-plane field is swept until the magnetization is saturated in-plane. The maximized signal collected for θ = 90° and φ = 45° (Fig. S5(b),(c)) shows the field required for saturation, which is related to the magnetic anisotropy energy (MAE) by the following equation:

$$K_u = \frac{\mu_0 H_K}{2M_s} \tag{27}$$

Then, contributions to anisotropy from known sources (magnetostatic, magnetoelastic, and magnetocrystalline) were subtracted from the total energy to isolate the additional anisotropy due to cation ordering according to this equation:

$$K_{U,eff} = \frac{9}{4}\lambda_{111}c_{44}\left(\frac{\pi}{2} - \beta\right) - \left(\frac{\mu_0}{2}\right) M_s^2 + \frac{K_1}{12} + K_{MT} \tag{28}$$

We account for magnetocrystalline anisotropy as the linear interpolation of the crystalline contributions of the endmembers, which is very small compared to other anisotropy terms. $K_1/12$

= -3800/12 = 317 J/m^3 for EuIG and -5800/12 = 483 J/m^3 for TmIG. Measured properties and calculated anisotropies for each $(Eu_xTm_{1-x})_3$IG sample are given in Table S5.

Table S5. *Anisotropy Calculations*

x (100% EuIG)	Ms (kA/m)	H_K (mT)	$\lambda_{111}c_{44}$ (Pa)	β (°)	$K_{u,eff}$ (J/m^3)	K_{MS} (J/m^3)	K_{ME} (J/m^3)	K_{MT} (J/m^3)
0	129.8	70.7	-842600	90.36	4589	-10590	1188	3783
0.196	97.15	106.1	-641173	90.15	5152	-5930	3862	7220
0.503	112.6	348.5	-324906	89.83	19620	-7966	-2146	29730
0.613	109.5	388.9	-210976	89.72	21290	-7534	-2348	31170
0.905	82.60	237.1	89796.6	89.41	9792	-4287	2064	12020
1	88.80	70.71	187320	89.32	3139	-4955	5021	3073

Saturation field is extracted from SMR by numerically fitting the signal to a macrospin model, which determines the equilibrium magnetization direction for any applied field magnitude and direction[8]. Error is estimated to be the field step. The data and fit of a representative curve are shown in Fig. S5(c).

The magnetoelastic constants, $\lambda_{111}c_{44}$, were determined from SMR measurements of anisotropy on series of EuIG and TmIG films grown on (111) substrates with different lattice parameters, including GGG (a_s = 1.2376 nm), YSGG (a_s = 1.2426 nm), NGG (a_s = 1.2505 nm), GSGG (a_s = 1.2554 nm)[4,9]. Fig. S6(a)(b) shows the strain series of EuIG films. From the linear variation of anisotropy as a function of strain we find the value of $\lambda_{111}c_{44}$ by linear regression of this equation:

$$K_{U,eff} = \frac{9}{4}\lambda_{111}c_{44}\left(\frac{\pi}{2} - \beta\right) - \left(\frac{\mu_0}{2}\right)M_s^2 + \frac{K_1}{12} \tag{29}$$

From this analysis, λ_{111} for EuIG and TmIG are found to be (2.45 ± 0.8)×10^{-7} and (-1.1 ± 0.1) ×10^{-6} if c_{44} is taken to be that of YIG (766 GPa)[10]. The calculated magnetostriction coefficients are about a factor of five lower than the reported values for these materials. This could indicate a non-ideal stoichiometry or c_{44} could deviate from the bulk value for YIG.

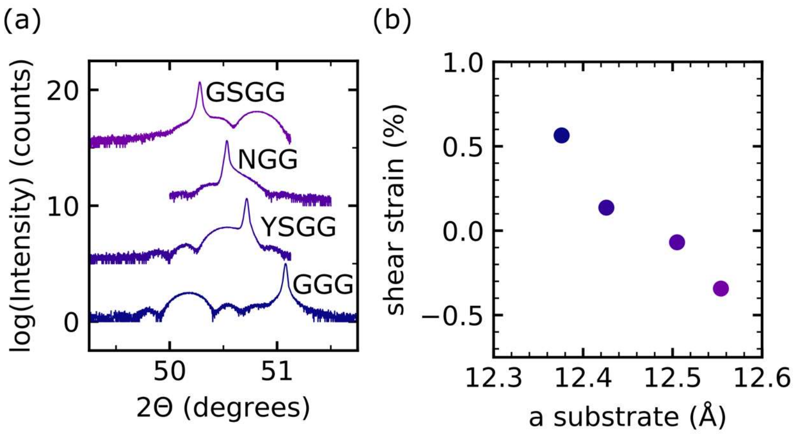

Fig. S6. Magnetostriction Determination. (a) (444) symmetric XRD scans of EuIG grown on GSGG, NGG, YSGG, and GGG. (b) Calculated strain in the EuIG films as a function of substrate lattice parameter.

By interpolating the values of $\lambda_{111}c_{44}$ for the mixed EuTmIG, and using Ms measured directly from the films, we avoid the use of literature values in the calculation of anisotropy and minimize associated errors. Strain, β, is calculated from XRD fitting, as described in Supplementary Note 2.

***Supplementary Note 5**: Derivation of uniaxial anisotropy from three tilted anisotropies and consequences*

Assuming each anisotropy axis unit vector $\hat{u}_i$ can be written in the Cartesian basis using spherical coordinates, as $(\sin\theta' \cos\phi', \sin\theta' \sin\phi', \cos\theta')$, while the magnetization vector is $\vec{m} = \sin\theta\cos\phi, \sin\theta\sin\phi, \cos\theta$, and the uniaxial anisotropy energy contribution is proportional to the dot product of the two quantities squared:

$$\varepsilon_j = \left(\sin\theta\sin\theta_j'\left(\cos\phi\cos\phi_j' + \sin\phi\sin\phi_j'\right) + \cos\theta\cos\theta_j'\right)^2 \tag{30}$$

For simplicity, we name certain terms with variables a and b which are equal for each axis, apply a trigonometric identity, and carry out the square.

$$\varepsilon_j = a^2\cos^2\left(\phi - \phi_j'\right) + 2ab\cos\left(\phi - \phi_j'\right) + b^2 \tag{31}$$

Now, assuming there are n such axes, all with the same polar angle θ', with azimuthal angles evenly distributed, i.e. $\phi_j' = \frac{2\pi j}{n}$, the total energy is

$$\varepsilon = \sum_{x=0}^{n-1} \varepsilon_x = a^2 \sum_{x=0}^{n-1} \cos^2\left(\phi - \frac{2\pi x}{n}\right) + 2ab \sum_{n=0}^{n-1} \cos\left(\phi - \frac{2\pi x}{n}\right) + nb^2 \tag{32}$$

The second term cancels out, proven below using complex numbers.

$$\sum_{x=0}^{n-1} \cos\left(\phi - \frac{2\pi x}{n}\right) = \sum_{x=0}^{n-1} \mathrm{Re}\{e^{2\pi xi/n} e^{-i\phi}\} = \mathrm{Re}\left\{e^{-i\phi} \sum_{x=0}^{n-1} e^{2\pi xi/n}\right\} \tag{33}$$

$$\sum_{x=0}^{n-1} e^{2\pi xi/n} = e^{2\pi i/n} \left(\frac{1 - e^{2\pi i}}{1 - e^{2\pi i/n}}\right) \tag{34}$$

But we can write the sum as a finite geometric series, and the numerator cancels out, since $e^{2\pi i} = 1$, thus the second sum in the energy equation is zero.

For the first term, we invoke the trigonometric transformation $\cos^2 z = {}^1\!/_2\,(1 + \cos 2z)$.

$$\sum_{x=0}^{n-1} \cos^2\left(\phi - \frac{2\pi x}{n}\right) = \frac{1}{2} \sum_{x=0}^{n-1} \left(1 + \cos 2\left(\phi - \frac{2\pi x}{n}\right)\right) \tag{35}$$

$$= \frac{n}{2} + \frac{1}{2} \mathrm{Re}\left\{e^{-2i\phi} \sum_{x=0}^{n-1} e^{4\pi xi/n}\right\}$$

By the same logic, the geometric sequence term cancels out, thus we are left with $a^2 \frac{n}{2}$. The final energy expression for n axes becomes:

$$\varepsilon = \frac{n}{2} \sin^2\theta \sin^2\theta' + n \cos^2\theta \cos^2\theta' \tag{36}$$

The system has three important angles – 1) the vertical axis which is the symmetry axis of the cone drawn out by the anisotropy directions (set to be the z-axis, $\theta = 0$), 2) the magnetization angle and 3) the anisotropy cone angle. Thus, both azimuthal angles are important and appear in the expression.

Considering the anisotropy cone angle fixed, we can write the energy:

$$\varepsilon = \frac{n}{2}(1-\alpha^2)(1-\cos^2\theta) + n\alpha^2\cos^2\theta = \frac{n}{2}[(1-\alpha^2) + (3\alpha^2-1)\cos^2\theta] \quad (37)$$

In the system, average energy is determined by ε/n, and is uniaxial in nature.

Description of the planes of order are shown in Fig. S7.

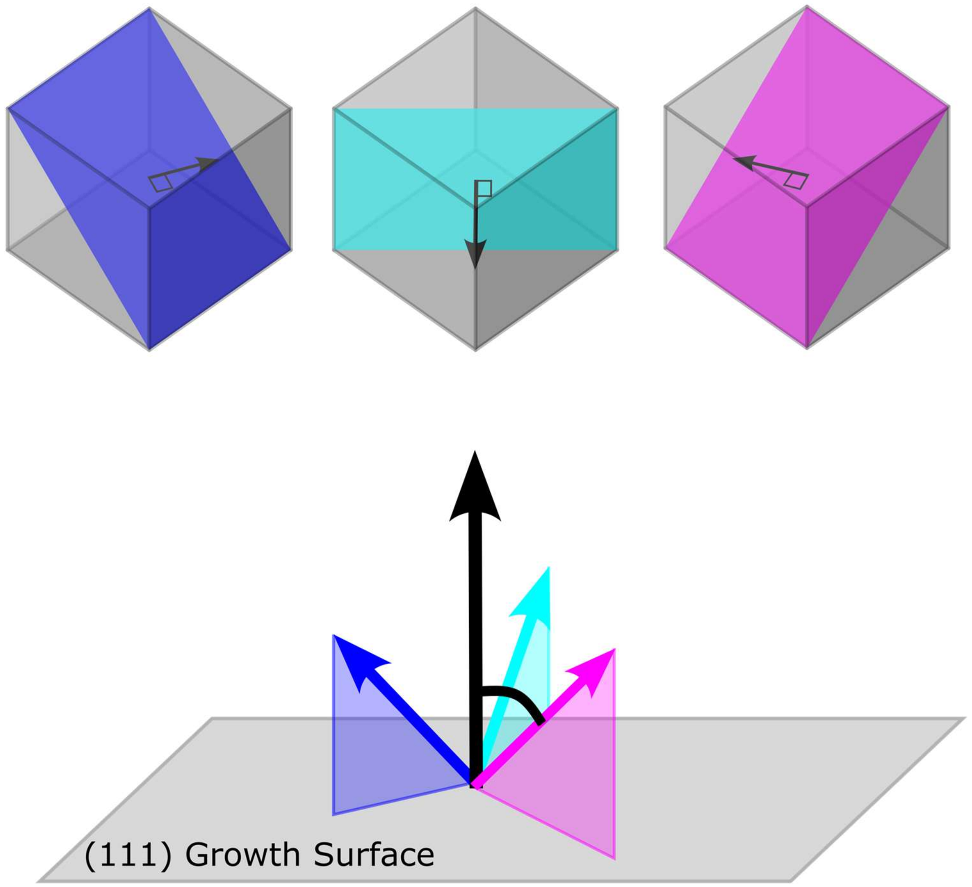

Fig. S7. Views of the individual tilted anisotropy axes for the variants of the ordered (111) REIG system (top and side views).

__Supplementary Note 6__: Scanning transmission electron microscopy (STEM)

It is essential to consider the role of the symmetry-allowed variants of the cation-ordering in order to analyse the structure and properties of the film. As described in Supplementary Note 5, for a (111) film an individual variant of the site order would yield a tilted anisotropy whereas the combination of the three variants produces PMA. Variants also affect the interpretation of XRD and STEM measurements. Fig. S8 illustrates variants for the case (111) and (110) films. In (a) an ordered unit cell of one variant of the (111) case is shown with a reference plane in red; red and blue balls represent the inequivalent c-sites. In Fig. S8(c), multiple unit cells are shown for the (111) ordering, each corresponding to one of the three variants. Because of the superposition of the three variants, there is no global order in the c-sites that can be measured by EDS unless the spatial extent of the variants is large compared to the sample. In contrast, for the case of (110) order, Fig. S8(b) shows one variant in which red, blue and purple balls represent the three sets of inequivalent c-sites α, β, γ. Fig. S8(d) shows a film consisting of the two variants. If we image along one specific zone axis it is possible to identify the order by EDS: we can distinguish red from blue/purple sites, since long range ordering of the red site motif prevails, but blue sites cannot be distinguished from purple.

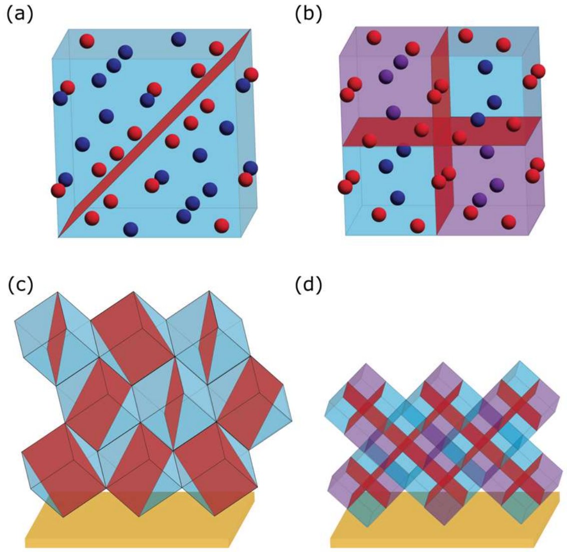

Fig. S8. Microstructural consequences of order variants. (a) (111) order scheme, showing dodecahedral sites only. (b) (110) order scheme, showing dodecahedral sites only. (c) Stacked order variants of the (111) scheme. (d) Stacked order variants of the (110) scheme.

The (111) EuTmIG films follow the scheme of Fig. S8a,c. STEM along the [$\bar{1}$10] in-plane zone axis, Fig. S9, shows no elementally resolved site ordering because the sample consists of all three variants. The intensity in a STEM image results from averaging all the atoms in the column along the beam direction. Collecting information through all three variants averages out any compositional ordering through the thickness of the TEM lamella. Indeed, we can conclude that the correlation length of these variants is on the scale of a few unit cells or less. The lamella of the (111)-oriented film was ~10 nm thick, but we do not observe cation order, indicating that the size of the variants is smaller than 10 nm, and may be on the scale of one or a few unit cells (the unit cell size is ~1.2 nm).

This precludes visualizing the *c* site order in (111) garnet films unless we had a lamella that is thinner than the correlation distance of the site-order.

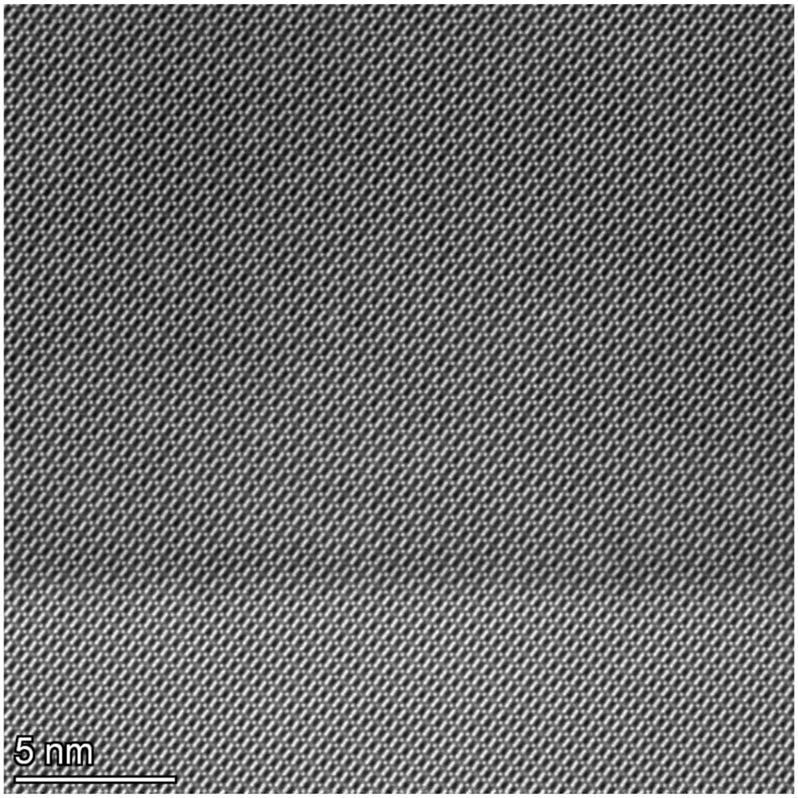

Fig. S9. STEM HAADF image of (111) oriented EuTmIG/GGG along the [$\bar{1}$10] in-plane zone axis, with substrate interface shown by a contrast change as Ga (Z=31) is replaced by Fe (Z = 26).

Therefore, we detect ordering instead in a EuTmIG film with (110) growth surface, using a $[1\bar{1}1]$ in-plane zone axis. There are only two variants of the ordered structure due to the mirror plane, and columns of α (red balls) and β/γ (purple and blue balls) sites are visible along $[1\bar{1}1]$ as indicated in Fig. S8d. For this zone axis we can distinguish α from β+γ groups but not β from γ.

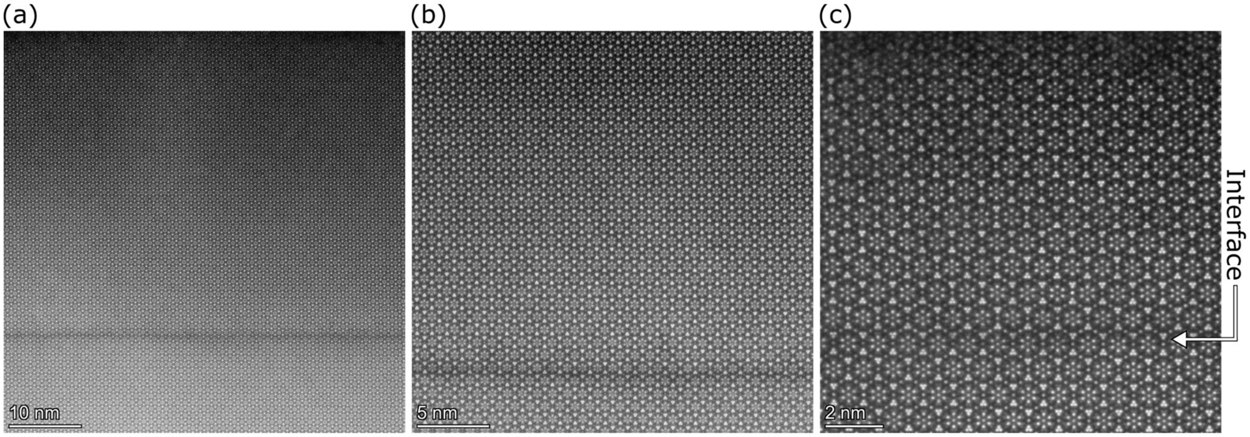

Fig. S10. $[1\bar{1}1]$ zone axis of (110) grown EuTmIG on GGG. (a) View of interface with 10 nm scale bar. (b) View of interface with 5 nm scale bar. (c) View of interface with 2 nm scale bar. In each figure, the interface appears darker.

STEM imaging (Fig. S9, S10 a-c) shows that the films are coherent with the substrate. The interface in Fig. S10 shows as darker contrast which may indicate vacancies or lower Z elements. EDS of the (111) film interface indicates that the composition changes across approximately one unit cell (~1 nm) (Fig. S11).

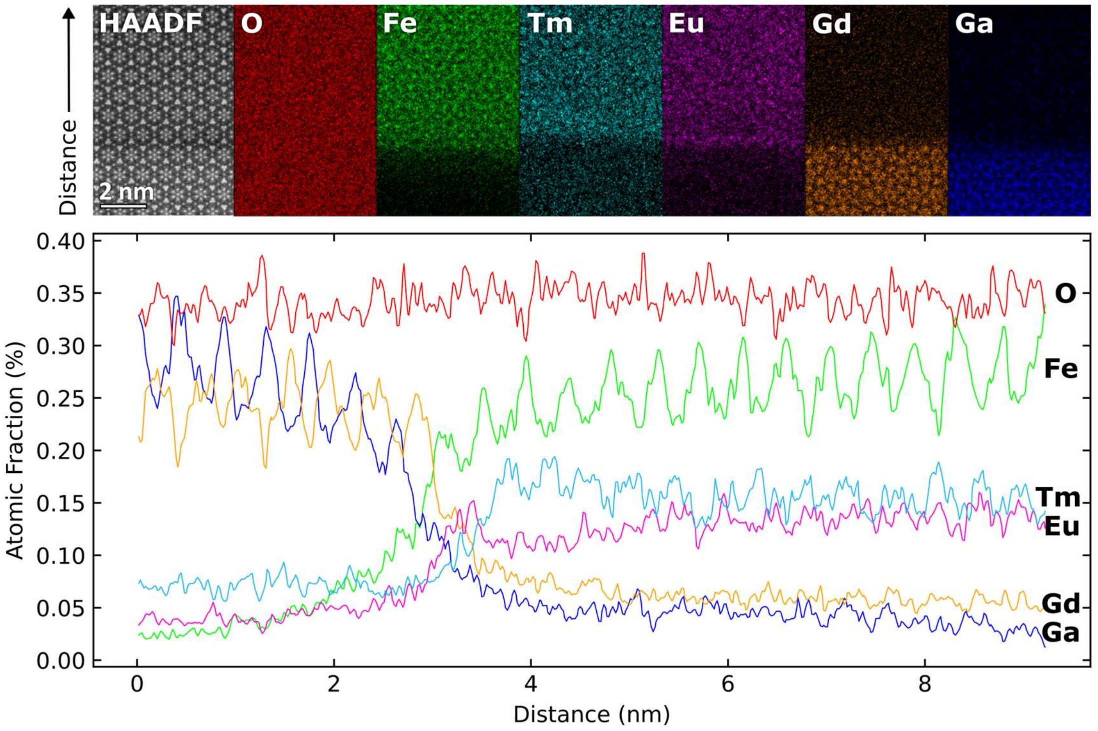

Fig. S11. EDS of the film-substrate interface.

For the (110)-oriented EuTmIG film, compositional analysis was carried out by EDS, analyzing the peaks of Eu, Tm, and Fe chosen to minimize overlap (Fig. S12). Then, non-linear principal component analysis was applied to reduce Poisson noise[11]. Lastly, the filtered image was slightly blurred with a Gaussian filter. Evidence of ordering can be seen even in the raw images (Fig. S12), but image processing makes the order immediately evident to the reader.

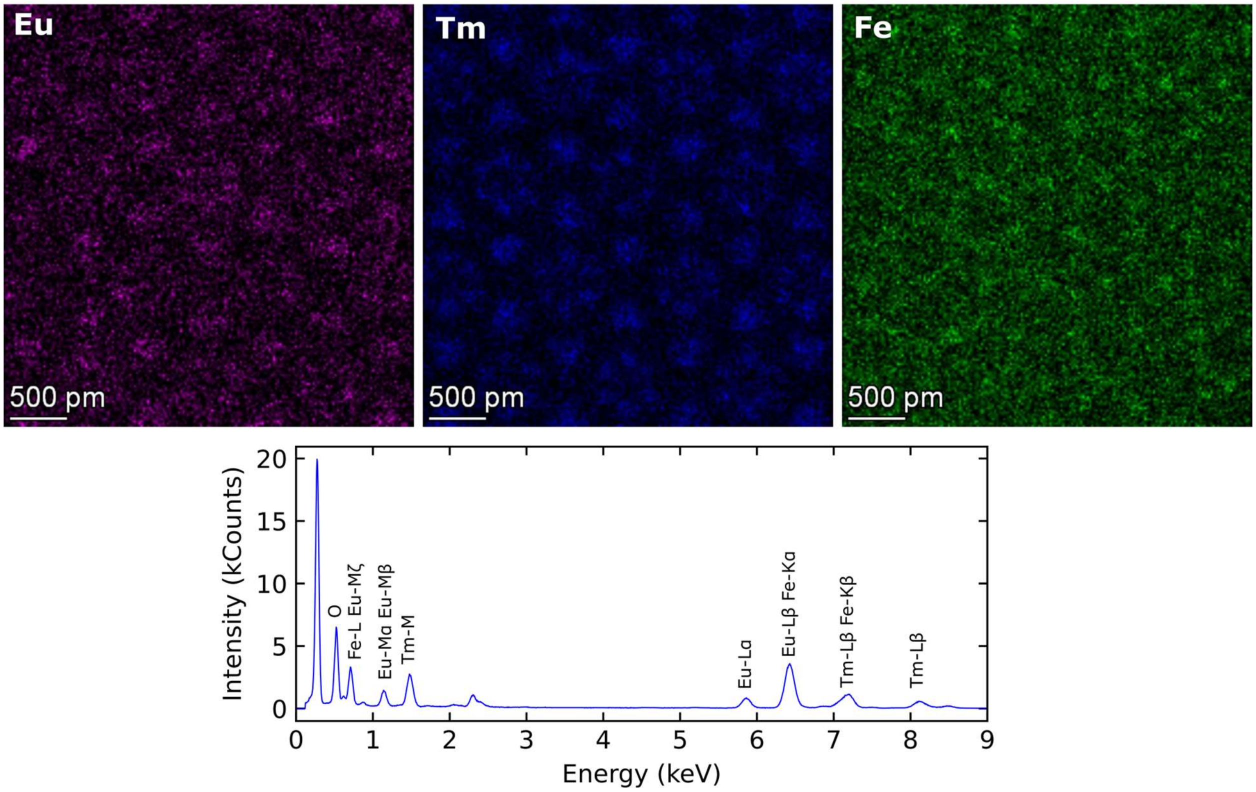

Fig. S12. Unprocessed EDS of Eu, Tm, and Fe in the ordered EuTmIG film and total EDS spectra.

Quantitative analysis of HAADF images of the film was also performed. The HAADF is sensitive to atomic number, Z, such that the averaged column intensities scale with approximately Z^2 and the number of atoms present in the column[12]. Tm peaks should be higher intensity than Eu, since $Z_{Tm} = 69$ and $Z_{Eu} = 63$.

First, a linear background subtraction was applied to reduce the intensity change due to the thickness gradient of the TEM lamella created during ion beam preparation. Then, atom columns were identified and fit using open-source code "Pycroscopy"[13]. Lastly, atom columns were masked with circles, and the intensity of each atom column was summed to minimize error in intensity measurement[14]. These atom columns were then binned into A, B, C, and D types, and averaged. Peak identification and an intensity histogram are shown in Fig. S13.

To examine the site occupancy of columns A,B,C and D, 1131 columns were identified. A and C columns both contain sites from the α group, but have different densities of atoms so different

HAADF intensities, with the D columns higher intensity than the A columns. B and C columns contain β and γ group sites, with C columns higher intensity than the B columns.

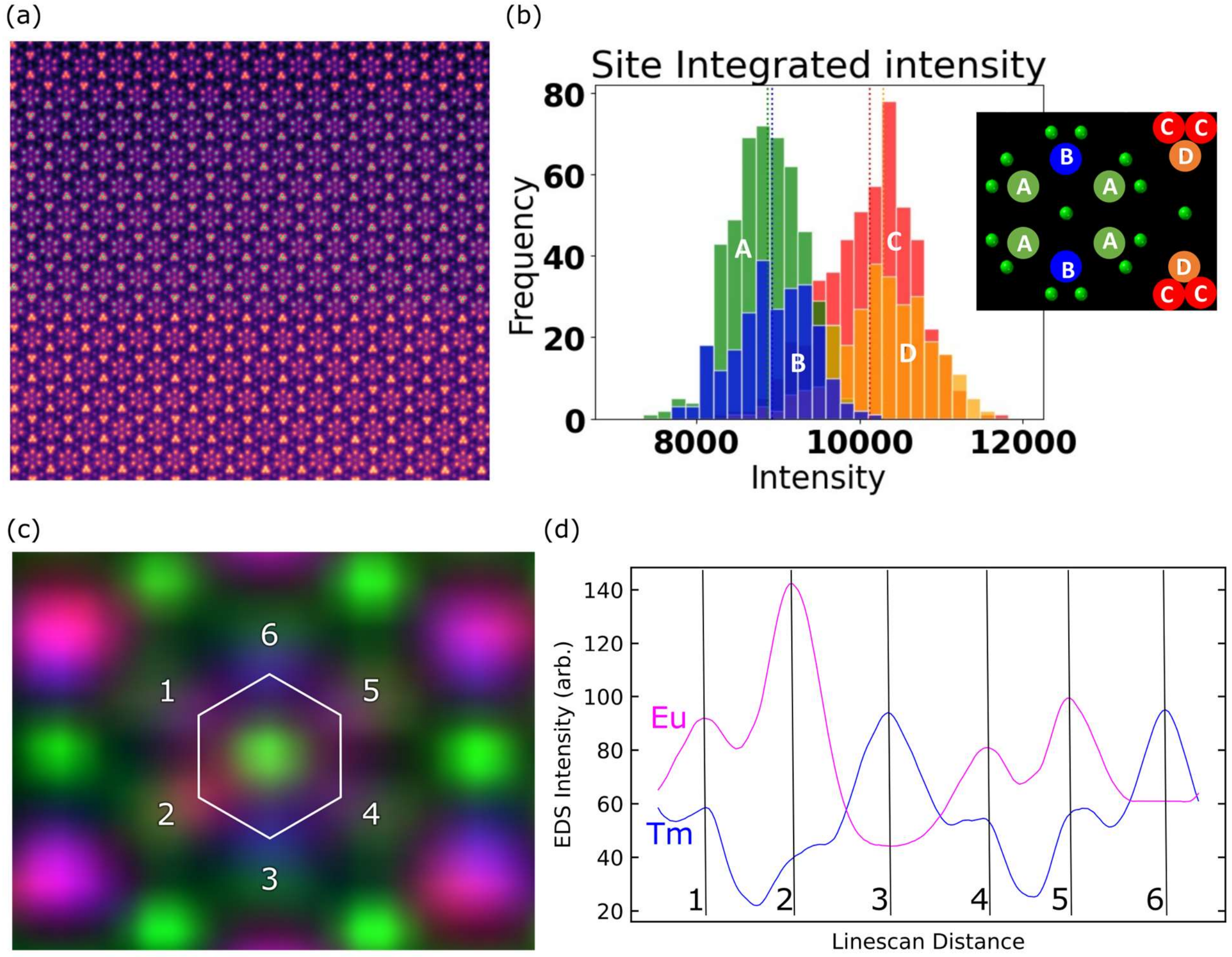

Fig. S13. Atom column intensity comparison. (a) selected atoms from a single image (b) histogram of atom column intensities for A, B, C, and D sites. (c) EDS of the (110) EuTmIG, showing site preference for Eu on A sites and Tm on B sites. (d) Extracted intensity line scan for Eu and Tm along the path of the hexagon in (c).

Recalling (Note 2) that there are twice as many α sites as $\beta+\gamma$ sites, we expect for a site-ordered EuTmIG with $x = 0.5$ that Tm should occupy all the $\beta+\gamma$ sites and ¼ of the α sites, and Eu should occupy ¾ of the α sites. The summed intensities were averaged for the two groups of atoms to give intensities that should be greater for the Tm-containing sites, as shown in Table S6.

Table S6. *Averaged atom column intensity and relevant statistics.*

Site (predicted element)	Averaged atom column intensities	Standard Deviation of the set	N
Eu	32,737	3,593	753
Tm	33,321	3,952	378

The analysis does not yield a statistically higher intensity for all the Tm-containing sites, which is inconsistent with the clear site-ordering observed in EDS. The discrepancy may be explained if there are cation vacancies (V_{RE}) or Fe antisite defects (Fe_{RE}) in the RE columns: even a small amount of these point defects would give a larger effect on column intensity than the Z-contrast between the Tm and Eu. Indeed, when we look at the Fe EDS, we see a slight indication that excess Fe could be preferring β and γ sites over α sites.

We also compared the Eu and Tm distributions locally in the hexagonal rings of A and B columns. Fig. S13(c,d) shows the analysis of one region of the sample with a hexagon of dodecahedral sites. Columns 1, 2, 4 and 5 contain sites from the α group and columns 3 and 6 contain sites from the β group. The line plot clearly shows that columns 3 and 6 contain the least Eu and most Tm, and the other columns have more Eu and less Tm. The peaks are not exactly the same for columns 3 and 6 (or for columns 1, 2, 4 and 5) because the column contains ~20 atoms and there would be a statistical fluctuation in the number of each atom in the columns, but the overall trend is clear and was also found in other sections of the image.

Concerning the role of defects, we did not observe any dislocations across the entire visible lamellae of (111) or (110)-oriented films. This is consistent with many other TEM investigations of epitaxial RE, Bi or Y garnets in our prior work[15,16]. Furthermore, the strain in the films does not relax even for thicknesses of 10s of nm, according to the RSM data, suggesting that dislocations do not form. Hence we do not believe dislocations play a major role in the site occupation or growth-induced anisotropy. Fe^{2+} is another possible point defect arising from oxygen deficiency. We performed X-ray absorption spectroscopy on TbIG films in a prior work[16] which showed that amount of Fe^{2+} was less than about 3%. Further, we would expect all the films to show similar amounts of Fe^{2+} or other point defects such as vacancies because they

were grown under similar conditions, so the dramatic differences in anisotropy between end-members and the mixed RE garnet is attributed to the RE ordering and not to Fe^{2+} or vacancies.

Supplementary Note 7: *Density Functional Theory (DFT) Calculations*

In DFT calculations, both the choice of pseudopotential and the initialization of magnetic moments significantly affect how the electronic structure calculation converges. Here the pseudopotentials were chosen to be the same pseudopotentials as those of Nakamoto et al.[17] The number of valence electrons considered for Fe ions is eight ($3d^6$ $4s^2$) and for O ions is six ($2s^2$ $2p^4$). Of the pseudopotentials chosen for the PBE functional, those which included the most valence electrons (including *f*-electrons in the valence) were selected: Eu ($4f^7$ $5s^2$ $5p^6$ $6s^2$) and Tm ($4f^{13}$ $5s^2$ $5p^6$ $6s^2$). In addition, following the procedure outlined, the magnetic moments were initialized such that the magnetization of the rare-earth ions is antiparallel to that of the tetrahedral Fe ions while being parallel to that of the octahedral Fe ions. The magnetic moments for each atom, as defined by the spin-polarized charge densities within the spheres defined by the Wigner-Seitz radius in the pseudopotential, were confirmed to be the same as previous reports from collinear calculations.

First, each structure was relaxed using collinear, spin-polarized methods described above. Then, to probe the magnetic anisotropy energy (MAE), non-collinear spin-polarized calculations with spin-orbit coupling were employed by rotating all spins according to different directions in the crystal. Given the computationally expensive nature of non-collinear calculations with spin-orbit coupling, non-self consistent calculations were performed, whereby the charge density is kept constant during the entire electronic minimization. These non-self consistent calculations were initialized with a charge density from a collinear calculation with sufficient sampling of the Brillouin zone (4x4x4 k-point grid).

Structural relaxation of the cation-ordered unit cell shows that for (111) type ordering, the symmetry of dodecahedral and octahedral bond angles is reduced, as listed in Table S7. That is, for the mixed species, local distortions of the atom sites, including bond angles and lengths, are changed. The local distortions cause a change in orbital overlap of the magnetic elements in a crystal, thus changing the strength of the magnetic exchange. In this way, distortion of Fe sublattices that occurs even when the *c* sites are filled with non-magnetic species could contribute to magnetic anisotropy and promote PMA, as seen for BiYIG.[18]

Structural relaxation for the three garnets (mixed garnet and two end members) was performed, accounting for the energy, per-atom forces, and stress computed by spin-polarized, collinear calculations at each update of the atomic positions. Then, a final spin-polarized, non-collinear calculation was performed on this static relaxed structure to evaluate the energy with a specific magnetization orientation. Including energy changes due to magnetostriction would have required accounting during structural relaxation for the energy, per-atom forces, and stress that arise from orientation of the magnetization. Such a method for structural relaxation was deemed prohibitively expensive since it would entail minimizing the energy and ensuring per-atom forces approach zero with spin-polarized, non-collinear electronic structure calculations performed at each update of the atomic positions rather than merely at the final step at the end of the structural relaxation. As a result, the calculation method presented does not include this magnetostriction and elastic energies. However, the mixed garnet has a smaller magnetostriction coefficient than the end members, and magnetostriction would therefore be unlikely to account for the larger anisotropy energy observed for the mixed garnet by DFT.

Table S7. *Bond Length and Angle Distortion*

O-Eu-O bond angles (°)		O-Tm-O bond angles (°)	
EuIG	EuTmIG	TmIG	EuTmIG
67.68	68.24	68.57	67.94
67.68	68.24	68.57	67.94
67.68	68.38	68.57	68.13
67.68	68.38	68.57	68.13
72.46	70.14	70.18	70.81
72.46	70.14	70.18	70.81
72.46	72.34	70.18	72.16
72.46	72.34	70.18	72.16
73.52	73.63	74.63	74.42
73.52	73.63	74.63	74.42
74.65	73.74	75.81	74.64
74.65	77.32	75.81	74.64
96.13	96.46	97.33	96.78
96.13	96.46	97.33	96.78
96.13	96.8	97.33	96.79
96.13	96.8	97.33	96.79
107.11	106.43	106.29	106.13
107.11	106.43	106.29	108.33
110.03	109.48	108.09	108.61
110.03	109.48	108.09	108.76
125.05	124.44	125.22	124.4
125.05	124.44	125.22	124.4
125.05	125.87	125.22	125.58
125.05	125.87	125.22	125.58

O-Fe_{oct}-O bond angles (°)		
EuIG	TmIG	EuTmIG
84.83	82.77	82.23
84.83	82.77	82.23
84.83	82.77	84.32
84.83	82.77	84.32
84.83	82.77	85.08
84.83	82.77	85.08
95.17	97.23	94.92
95.17	97.23	94.92
95.17	97.23	95.68
95.17	97.23	95.68
95.17	97.23	97.77
95.17	97.23	97.77

O-Fe_{tet}-O bond angles (°)		
EuIG	TmIG	EuTmIG
99.67	98.67	97.79
99.67	98.67	100.85
114.58	115.13	114.5
114.58	115.13	114.63
114.58	115.13	114.73
114.58	115.13	115.19

Eu-O bond lengths (Å)		Tm-O bond lengths (Å)	
EuIG	EuTmIG	TmIG	EuTmIG
2.38	2.37	2.32	2.31
2.38	2.37	2.32	2.31
2.38	2.41	2.32	2.32
2.38	2.41	2.32	2.32
2.48	2.45	2.38	2.40
2.48	2.45	2.38	2.40
2.48	2.46	2.38	2.42
2.48	2.46	2.38	2.42

Fe_{oct}-O bond lengths (Å)		
EuIG	TmIG	EuTmIG
2.01	2.00	2.00
2.01	2.00	2.00
2.01	2.00	2.01
2.01	2.00	2.01
2.01	2.00	2.01
2.01	2.00	2.01

Fe_{tet}-O bond lengths (Å)		
EuIG	TmIG	EuTmIG
1.87	1.85	1.86
1.87	1.85	1.86
1.87	1.85	1.86
1.87	1.85	1.86

Supplementary Note 8. *Simulations of X-ray Diffraction (XRD) from ordered garnets*

Modelling the proposed order schemes was particularly insightful as to the understanding of measured X-ray diffraction in our films, especially for the observation of the unique (110) ‘superlattice’ peaks forbidden for the disordered garnet structure. As noted from Callen’s formulation of growth-induced anisotropy, cation order reduces the symmetry of the unit cell. Thus, the diffraction must change to reflect this order. Powder diffraction was simulated using CrystalMaker 11 and CrystalDiffract 7 for the cases of GGG, a typical substrate with no cation order, and for $Eu_{1.5}Tm_{1.5}Fe_5O_{12}$ with ordering corresponding to growth along three different directions (Fig. S14). While all of our measurements are on epitaxial thin films, powder diffraction simulation can still inform the location of key peaks in reciprocal space.

Our simulations show that Eu (Z=63) and Tm (Z=69) are sufficiently different in atomic number to produce a measurable peak intensity in the ordered state. Importantly, as the symmetry of the growth plane decreases from (111) → (110) → (112), more ‘superlattice’ peaks unique to the RE site-order emerge. The specific peak we measure (in asymmetric geometry) in the (111) grown EuTmIG to prove the RE order is the (110) peak, shown around $2\theta = 10°$.

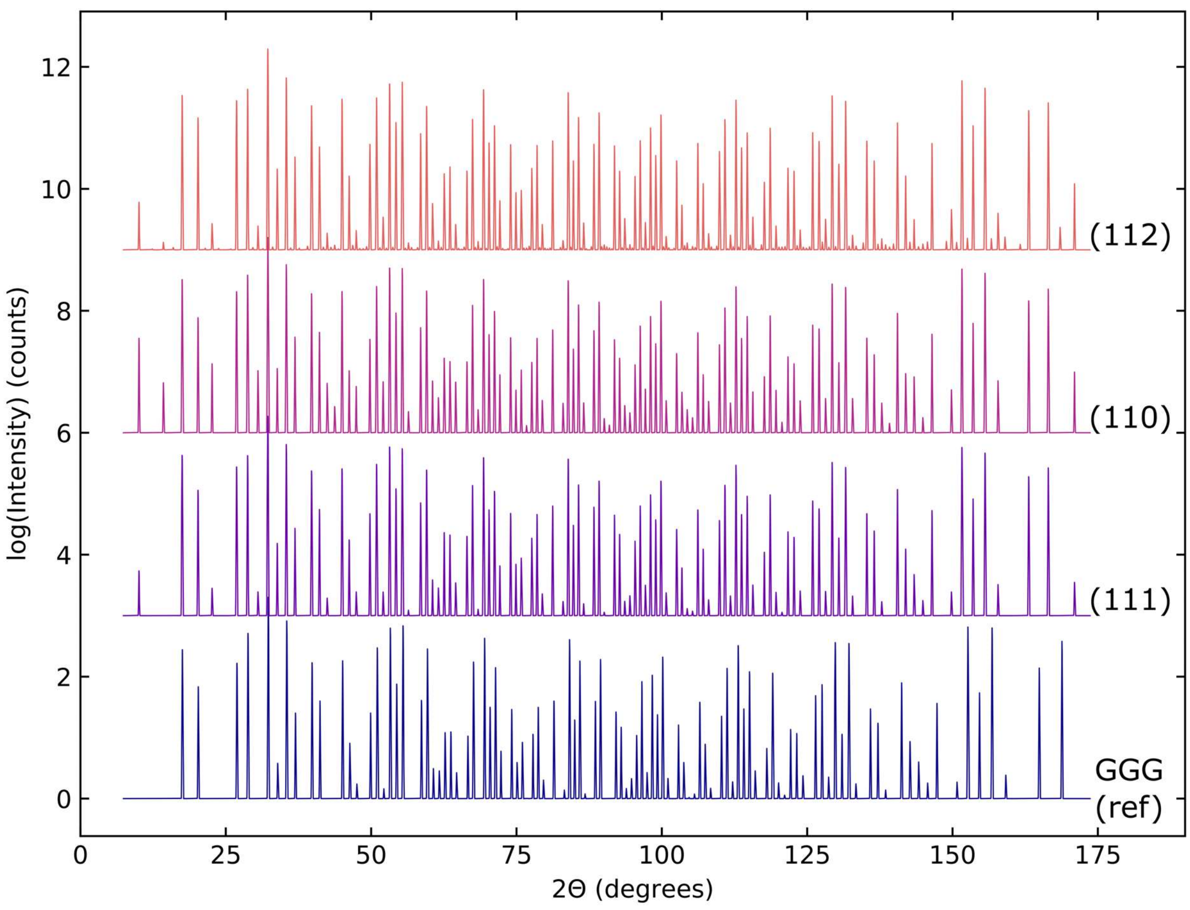

Fig. S14. Simulated powder diffraction for the substrate (GGG) and for $Eu_{1.5}Tm_{1.5}Fe_5O_{12}$ with (111), (110), and (112) type ordering (source: CuKα). The peak around 10˚ is a clear indicator of dodecahedral site order.

Fig. S15 shows that for a site-ordered unit cells with fixed RE composition ($Eu_{1.5}Tm_{1.5}Fe_5O_{12}$), as the degree of ordering of the RE on distinguishable sites increases, the intensity of the (110) order peak also increases with a quadratic dependence. The peak intensity depends on the structure factor which varies with the difference in atomic number of the ordered RE cations. For the completely mixed case, there is no difference in the average atomic number on each site, so no peak is seen.

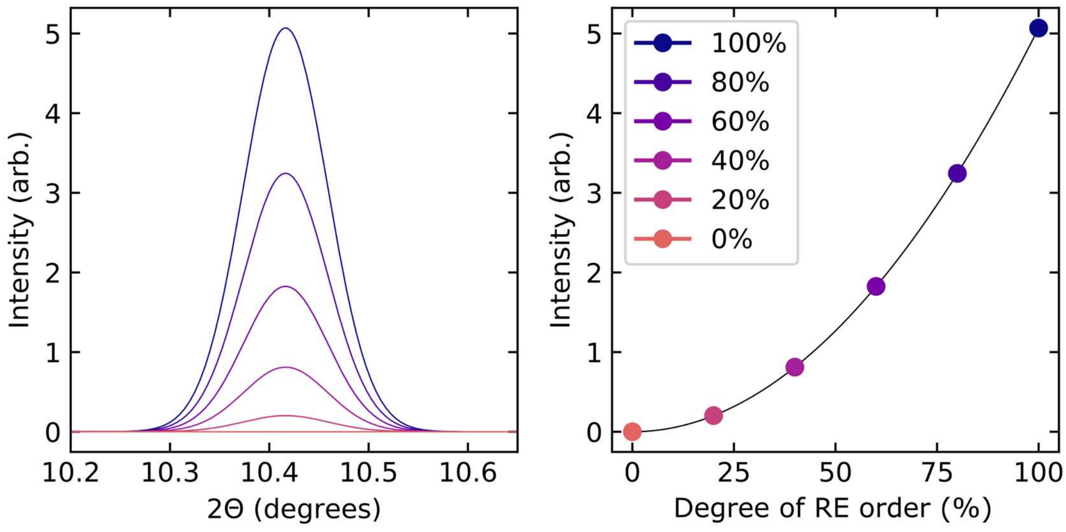

Fig. S15. Simulated powder diffraction for the (110) superlattice peak of $Eu_{1.5}Tm_{1.5}Fe_5O_{12}$ with varied degree of order in the unit cell (source: CuKα).

Lastly, to explain the observation of weak (110) peaks in the asymmetric scans of endmember EuIG and TmIG films grown on (111) GGG (Fig. 3), we consider the effects of point defects. Generally, removing atoms from a unit cell reduces the symmetry of the cell, resulting in the appearance of structurally forbidden peaks. Fig. S16 shows the consequence of removing a single oxygen atom from the unit cell of GGG, for example. Even the removal of one atom in 160 produces sufficient change in the structure factors to produce peaks which were absent in the perfect crystal.

We do not expect many defects in the Czochralski-grown GGG substrates, and the (110) peak of the GGG in Fig. 3 is absent. However, YIG or REIG films growth by pulsed laser deposition often exhibit non-ideal cation stoichiometry or oxygen deficiency.[16] The presence of a weak (110) peak for the endmembers EuIG and TmIG is readily explained by such point defects. Point defects are also expected to contribute to the (110) peaks in the EuTmIG film, but the much greater intensity of the peak is attributed to the Eu/Tm site ordering.

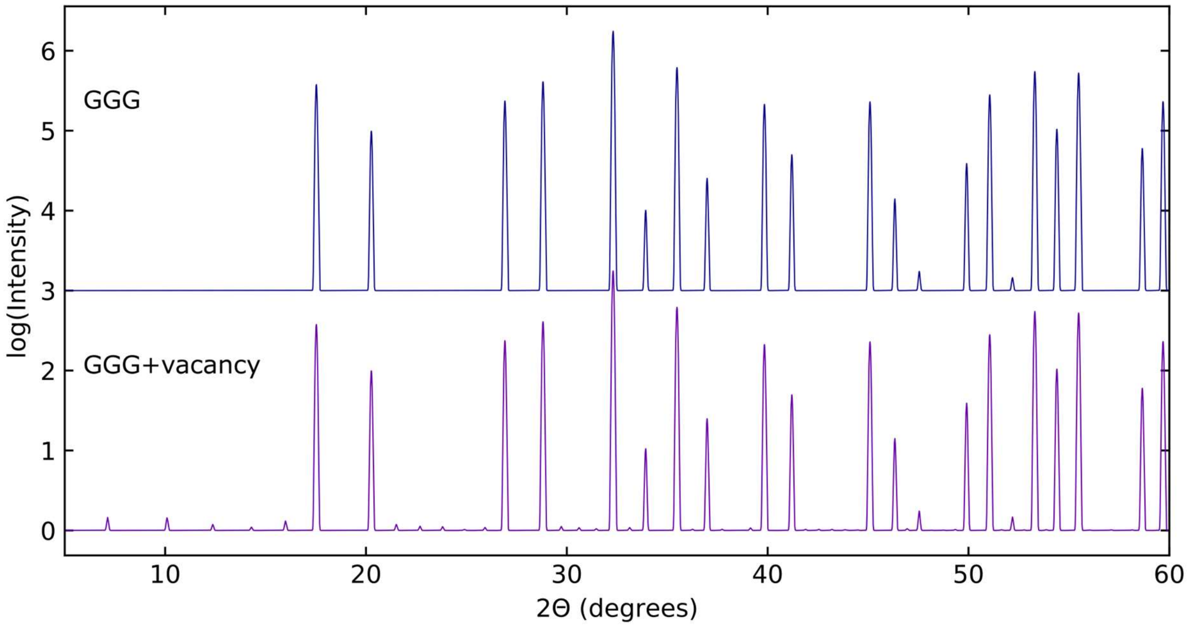

Fig. S16. Simulated powder diffraction of gadolinium gallium garnet in the perfect state (upper panel) and with one oxygen atom removed from the unit cell, forming a vacancy (lower panel).

Supplementary Note 9. *Relationship between XRD and STEM data for verification of cation order*

Relating the TEM evidence of site ordering from the (110)-oriented film and the XRD evidence of site ordering from the (111)-oriented film is critical to verify the existence of cation order in mixed RE-garnet films and support the conclusions put forth in this paper. For (111)-oriented films, XRD is the only method used to observe rare earth ordering in REIGs, since the presence of symmetry-related variants throughout the volume of the film precludes the use of electron microscopy to determine order (Supplementary Note 6). Therefore, as discussed in the main text, direct imaging by elementally resolved TEM was carried out on a lower symmetry (110) oriented film for absolute proof of ordering. However, it is also possible to use XRD methods to verify cation ordering in (110)-oriented films.

Fig. S17a shows the results of performing symmetric out-of-plane 2θ-ω scans on a (110)-oriented end-member EuIG film and a EuTmIG (x~0.5) (110)-oriented film, both of which were

grown concurrently with the (111) EuTmIG films of Fig. 2. The {110} set of reflections are clearly visible.

For the GGG substrate and the films, (220) and (440) reflections are present as expected since these even reflections are not systematically absent. The (110) peak (at 2θ = ~10°) is normally forbidden for the $Ia\bar{3}d$ cubic garnet crystal structure, but it is present for all samples. This is due to the well-known phenomenon of *umweganregung*, which causes the appearance of normally forbidden peaks in symmetric scans due to reasons other than symmetry reduction in the crystal (Fig. S17b).[19] The presence of the *umweganregung* peak prohibits us from using the (110) reflection to verify cation order in these films, since it is also present in the substrate. [It is important to note, however, that the (110) peak can still be used to diagnose order in the (111) films as shown in Fig. 2 because it is collected in a skew geometry, which reduces the *umweganregung* peak.]

In contrast, the higher order reflection, (330), is much less prone to *umweganregung.* This peak is therefore a good diagnostic of cation order. We note that (330) peaks exist for the EuIG and EuTmIG films on GGG, but not the uncoated GGG substrate (Fig. S17c). Moreover, the d-spacings of these (330) peaks are consistent with the higher lattice parameter of the EuIG compared to the EuTmIG, unlike the (110) peaks, giving us confidence that the (330) peaks are not *umweganregung* peaks from the substrate. The (330) peak of the EuTmIG is significantly higher than that of the EuIG. We believe that the EuIG shows some (330) intensity due to point defects (Supplementary Note 8), but the higher intensity of the EuTmIG peak is a result of RE site ordering. Thus, we can verify cation site order in the (110)-oriented EuTmIG from both XRD and STEM.

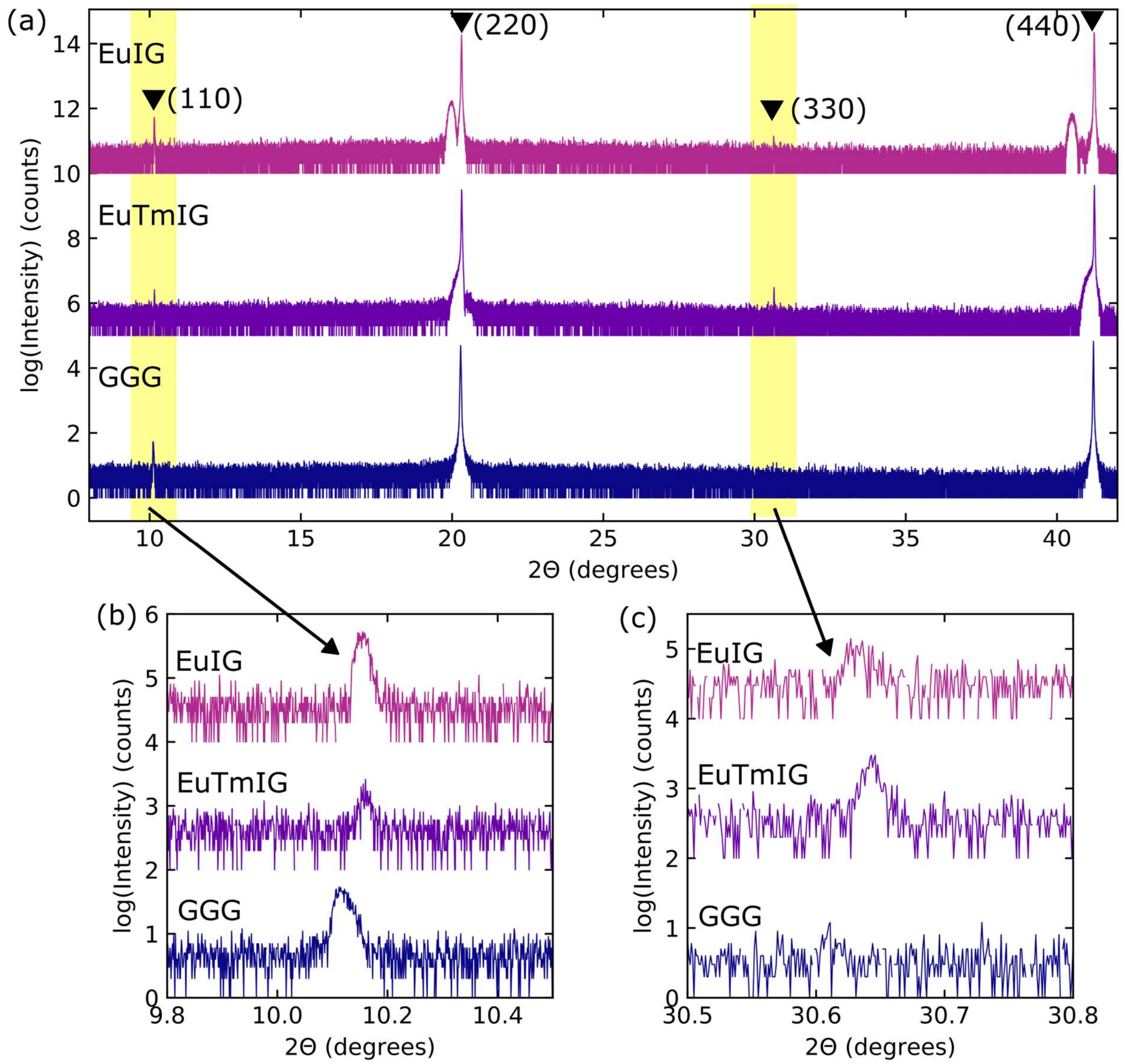

Fig. S17. Symmetric XRD scans for (110) films. (a) Symmetric 2θ-ω scans of EuIG and EuTmIG films grown on (110) GGG, compared to a bare (110) GGG substrate. (b) (110) reflections of the EuIG and EuTmIG films, compared to that of the bare (110) GGG substrate. (c) (330) reflections of the EuIG and EuTmIG films, compared to that of the bare (330) GGG substrate.

References

1. Callen, H. On growth-induced anisotropy in garnet crystals. *Materials Research Bulletin* **6**, 931–938 (1971).
2. Callen, H. Growth-Induced Anisotropy by Preferential Site Ordering in Garnet Crystals. *Appl. Phys. Lett.* **18**, 311–313 (1971).
3. Akselrad, A. & Callen, H. Growth-Induced Noncubic Anisotropy Arising from the Tetrahedral Sites in Garnets. *Appl. Phys. Lett.* **19**, 464–466 (1971).
4. Eschenfelder, A. H. *Magnetic Bubble Technology*. (Springer-Verlag, 1980).
5. van Vleck, J. H. On the Anisotropy of Cubic Ferromagnetic Crystals. *Phys. Rev.* **52**, 1178–1198 (1937).
6. Bhoi, B. *et al.* Observation of enhanced magnetic anisotropy in PLD YIG thin film on GGG (1 1 1) substrate. *Journal of Magnetism and Magnetic Materials* **483**, 191–195 (2019).
7. Chen, Y.-T. *et al.* Theory of spin Hall magnetoresistance. *Phys. Rev. B* **87**, 144411 (2013).
8. Song, Y., Kaczmarek, A. C., Beach, G. S. D. & Ross, C. A. Engineering an easy-plane anisotropy in an epitaxial europium iron garnet (110) film. *Phys. Rev. Mater.* **7**, 084407 (2023).
9. Fakhrul, T. *et al.* Influence of substrate on interfacial Dzyaloshinskii-Moriya interaction in epitaxial $Tm_3Fe_5O_{12}$ films. *Phys. Rev. B* **107**, 054421 (2023).
10. Landolt-Börnstein, New Series, K. H. Hellwege (ed.), Group III: Crystal and Solid State Physics, Vol. 12: Magnetic and Other Properties of Oxides and Related Compounds, Part c: Hexagonal Ferrites. Special Lanthanide and Actinide Compounds. Springer-Verlag Berlin, Heidelberg, New York (1982).
11. Salmon, J., Harmany, Z. & Deledalle, C.-A. Poisson noise reduction with non-local PCA.

12. Hartel, P., Rose, H. & Dinges, C. Conditions and reasons for incoherent imaging in STEM. *Ultramicroscopy* **63**, 93–114 (1996).

13. Somnath, S. *et al.* USID and Pycroscopy -- Open frameworks for storing and analyzing spectroscopic and imaging data. Preprint at https://doi.org/10.48550/arXiv.1903.09515 (2019).

14. E, H. *et al.* Probe integrated scattering cross sections in the analysis of atomic resolution HAADF STEM images. *Ultramicroscopy* **133**, 109–119 (2013).

15. Caretta, L. *et al.* Interfacial Dzyaloshinskii-Moriya interaction arising from rare-earth orbital magnetism in insulating magnetic oxides. *Nat Commun* **11**, 1090 (2020).

16. Rosenberg, E. *et al.* Revealing Site Occupancy in a Complex Oxide: Terbium Iron Garnet. *Small* **n/a**, 2300824. (2023).

17. Nakamoto, R., Xu, B., Xu, C., Xu, H. & Bellaiche, L. Properties of rare-earth iron garnets from first principles. *Phys. Rev. B* **95**, 024434 (2017).

18. Gouéré, D. *et al.* Temperature-independent ferromagnetic resonance shift in Bi-doped YIG garnets through magnetic anisotropy tuning. *Phys. Rev. Mater.* **6**, 114402 (2022).

19. The intensity of forbidden reflections of pyrope: Umweganregung or symmetry reduction? *Zeitschrift für Kristallographie - Crystalline Materials* **210**, 645–649 (1995).
