# Supplementary material for: Atomic order of rare earth ions in a complex oxide: a path to magnetotaxial anisotropy
Source: Nat Commun. 2024 Jun 14;15:5083. doi: 10.1038/s41467-024-49398-4 (PMC11178793; doi:10.1038/s41467-024-49398-4)
Supplement: Supplementary file 4 — Lasing Reporting Summary [file 41467_2024_49398_MOESM4_ESM.pdf]

nature research

Corresponding author(s): Allison Kczmarek

Lasing Reporting Summary

Nature Research wishes to improve the reproducibility of the work that we publish. This form is intended for publication with all accepted papers reporting claims of lasing and provides structure for consistency and transparency in reporting. Some list items might not apply to an individual manuscript, but all fields must be completed for clarity.

For further information on Nature Research policies, including our data availability policy, see Authors & Referees.

Experimental design

Please check: are the following details reported in the manuscript?

1. Threshold

Plots of device output power versus pump power over a wide range of values indicating a clear threshold

☐ Yes
☒ No

We do not report claims of lasing in the manuscript for our material of interest.

2. Linewidth narrowing

Plots of spectral power density for the emission at pump powers below, around, and above the lasing threshold, indicating a clear linewidth narrowing at threshold

☐ Yes
☒ No

We do not report claims of lasing in the manuscript for our material of interest.

Resolution of the spectrometer used to make spectral measurements

☐ Yes
☒ No

We do not report claims of lasing in the manuscript for our material of interest.

3. Coherent emission

Measurements of the coherence and/or polarization of the emission

☐ Yes
☒ No

We do not report claims of lasing in the manuscript for our material of interest.

4. Beam spatial profile

Image and/or measurement of the spatial shape and profile of the emission, showing a well-defined beam above threshold

☐ Yes
☒ No

We do not report claims of lasing in the manuscript for our material of interest.

5. Operating conditions

Description of the laser and pumping conditions
Continuous-wave, pulsed, temperature of operation

☐ Yes
☒ No

We do not report claims of lasing in the manuscript for our material of interest.

Threshold values provided as density values (e.g. W cm^{-2} or J cm^{-2}) taking into account the area of the device

☐ Yes
☒ No

We do not report claims of lasing in the manuscript for our material of interest.

6. Alternative explanations

Reasoning as to why alternative explanations have been ruled out as responsible for the emission characteristics
e.g. amplified spontaneous, directional scattering; modification of fluorescence spectrum by the cavity

☐ Yes
☒ No

We do not report claims of lasing in the manuscript for our material of interest.

7. Theoretical analysis

Theoretical analysis that ensures that the experimental values measured are realistic and reasonable
e.g. laser threshold, linewidth, cavity gain-loss, efficiency

☐ Yes
☒ No

We do not report claims of lasing in the manuscript for our material of interest.

8. Statistics

Number of devices fabricated and tested

☐ Yes
☒ No

We do not report claims of lasing in the manuscript for our material of interest.

Statistical analysis of the device performance and lifetime (time to failure)

☐ Yes
☒ No

We do not report claims of lasing in the manuscript for our material of interest.
